# Effects of Pre-Slaughter Dietary Supplementation with a Phytogenic Additive Containing *Passiflora incarnata* and *Stevia rebaudiana* Bertoni Extracts on Oxidative Status, Inflammatory Responses, Carcass Quality, Meat Composition, and Bone Morphology in Broilers

**DOI:** 10.3390/ani16192980

**Published:** 2026-09-22

**Authors:** Esin Ebru Onbaşılar, Suzan Yalçın, Kıvılcım Ateş Çakıroğlu, Barış Batur, Sakine Yalçın, İhsan Berat Kılıçlı, Caner Bakıcı, Gülcan Avcıoğlu

**Affiliations:** 1 Department of Animal Husbandry, Faculty of Veterinary Medicine, Ankara University, 06110 Ankara, Türkiye; 2 Department of Food Hygiene and Technology, Faculty of Veterinary Medicine, Selçuk University, 42003 Konya, Türkiye; syalcin@selcuk.edu.tr; 3 Department of Research and Development, HasTavuk, 10600 Balıkesir, Türkiye; kivilcimates@hastavuk.com (K.A.Ç.); gulcanavcioglu@hastavuk.com (G.A.); 4 Department of Anatomy, Faculty of Veterinary Medicine, Ankara University, 06110 Ankara, Türkiye; barisbaturr@gmail.com (B.B.); vetcanerbakici@gmail.com (C.B.); 5 Animal Nutrition Science Association, 06420 Ankara, Türkiye; 6 Faculty of Veterinary Medicine, Ankara University, 06110 Ankara, Türkiye; kilicli571@gmail.com

**Keywords:** corticosterone, oxidative stress index, physiological resilience, vision grading system, computed tomography

## Abstract

Background: The final days before slaughter represent one of the most stressful periods in commercial broiler production, during which acute stress stimulates endocrine, oxidative, and inflammatory responses that may compromise animal welfare and slaughter outcomes. This study evaluated the effects of short-term dietary supplementation with a phytogenic additive containing *Passiflora incarnata* and *Stevia rebaudiana* extracts (Passiflora–Stevia phytogenic additive; PSPA) during the final five days before slaughter on carcass characteristics, physiological stress, oxidative status, inflammatory responses, tissue composition, and bone morphology in broilers. Methods: A total of 106,240 Ross 308 broilers were assigned to either a control group or a PSPA-supplemented group (1 kg/t) during the final five days before slaughter (days 38–42). Carcass characteristics, breast muscle and liver composition, serum corticosterone, inflammatory cytokines, oxidative stress biomarkers, and bone morphology using three-dimensional geometric morphometrics were evaluated. Results: PSPA supplementation significantly reduced serum IL-1β, IL-6, TNF-α, corticosterone, total oxidant status, and oxidative stress index, while increasing IL-10 concentrations (*p* < 0.05). Total antioxidant status was unaffected. After adjustment for pre-slaughter live weight, carcass weight was significantly higher in the PSPA group (*p* = 0.002), whereas carcass yield remained unchanged. PSPA-treated broilers also showed favorable numerical trends in carcass quality, including a higher proportion of Grade A carcasses and fewer carcass defects, although these differences were not statistically significant. Breast muscle and liver composition were unaffected, and only limited treatment-related differences were detected in bone morphology. Conclusions: Short-term PSPA supplementation improved physiological resilience by attenuating corticosterone secretion, oxidative stress, and inflammatory responses before slaughter. Although carcass quality was not significantly altered, the favorable numerical trends and improved physiological biomarkers suggest that targeted preslaughter phytogenic supplementation may represent a practical nutritional strategy to mitigate preslaughter stress in broiler chickens. Longer-term studies are warranted to determine whether these physiological benefits translate into improvements in bone characteristics and slaughter outcomes.

## Simple Summary

Commercial broilers experience considerable physiological stress during the final days before slaughter because of feed withdrawal, catching, transportation, and handling. This stress increases oxidative damage, inflammation, and corticosterone secretion, potentially compromising animal welfare and carcass quality. In this study, a phytogenic additive containing *Passiflora incarnata* and *Stevia rebaudiana* extracts was provided only during the last five days before slaughter to determine whether such a short-term intervention could improve physiological resilience under commercial conditions. More than 106,000 broilers were evaluated in a large-scale commercial trial. Supplementation reduced oxidative stress, lowered corticosterone and pro-inflammatory cytokine concentrations, and increased the anti-inflammatory cytokine IL-10, indicating an improved physiological status before slaughter. Birds receiving the phytogenic additive also had higher carcass weight after adjustment for preslaughter live weight and showed favorable numerical trends in carcass quality without affecting breast muscle or liver composition. Only minor changes were observed in bone morphology, which is consistent with the short duration of supplementation. These findings suggest that targeted phytogenic supplementation immediately before slaughter may represent a practical nutritional strategy to reduce physiological stress and improve resilience in commercial broiler production.

## 1. Introduction

The preslaughter period represents a distinct physiological challenge in commercial broiler production because feed withdrawal, catching, loading, transportation, and holding before slaughter expose birds to multiple acute stressors [1]. These procedures can alter physiological stress responses, including circulating corticosterone concentrations and metabolic status, and may subsequently influence carcass and meat quality characteristics [2,3]. The physiological stress response in poultry involves activation of neuroendocrine pathways and changes in oxidative homeostasis, with corticosterone serving as an important indicator of stress-related responses in birds [4,5].

The biological consequences of preslaughter stress are not limited to endocrine responses. Experimental studies in broilers have demonstrated that feed withdrawal, catching, crating, and transportation can alter energy metabolism and circulating metabolites, while preslaughter transport procedures can also affect corticosterone responses and postmortem muscle metabolism [1,3,6]. These findings indicate that the period immediately preceding slaughter represents a biologically dynamic phase in which endocrine and metabolic responses can change within a relatively short time frame. Consequently, nutritional interventions targeted specifically to this period may provide an opportunity to modulate physiological responses while minimizing the potential influence of long-term dietary supplementation on growth and tissue development.

Phytogenic feed additives are complex mixtures of plant-derived bioactive compounds, including phenolic acids, flavonoids, terpenoids, and other secondary metabolites that may influence oxidative, inflammatory, and other physiological processes [7,8,9]. Their biological activity is therefore not necessarily attributable to a single constituent but may reflect the contribution of multiple compounds present in the botanical matrix [5]. This multifunctional composition has led to increasing interest in phytogenic additives as nutritional modulators of oxidative, inflammatory, and other physiological responses in poultry. However, relatively few studies have specifically examined short-term phytogenic supplementation during the final days before slaughter, particularly in relation to endocrine, oxidative, and inflammatory responses under commercial production conditions.

Among botanical ingredients of interest, *Stevia rebaudiana* Bertoni contains diterpene glycosides, phenolic compounds, flavonoids, and other bioactive constituents with reported antioxidant and biological activities [10,11]. Experimental studies in poultry have reported several biological effects of stevia supplementation. Dietary stevioside has been investigated in relation to immune and physiological responses in broilers [12], while stevia-based supplementation has been reported to affect humoral immune responses [13] and gut health and cecal microbiota [14]. Under heat-stress conditions, dietary stevia and ginger extracts have been associated with changes in serum biochemical parameters in quails [15], whereas stevia leaf supplementation has been reported to influence antioxidant-related parameters, digestive enzyme activities, and meat quality in broilers [16]. More recently, dietary *S. rebaudiana* extract was reported to affect several meat quality characteristics in yellow-feathered broilers [17]. These findings indicate that *S. rebaudiana* may influence multiple physiological processes relevant to oxidative, immune, intestinal, and meat-quality-related responses in poultry.

*Passiflora incarnata* L. (passionflower) is another phytogenic plant characterized by a diverse profile of flavonoids and phenolic compounds and has been investigated for its antioxidant, neuroactive, and stress-related properties [18,19,20]. Experimental evidence suggests that some biological effects of *P. incarnata* may involve modulation of the γ-aminobutyric acid (GABA_A_) receptor system [21,22]. Importantly, experimental evidence from animal nutrition studies indicates that *P. incarnata* may influence physiological responses associated with stress. Dietary *P. incarnata* supplementation reduced aggressive behavior, ear lesions, and salivary cortisol concentrations in weaned pigs [23]. Although direct evidence for *P. incarnata* supplementation in broilers remains limited, these findings provide a biological basis for investigating its potential contribution to stress-related physiological responses in poultry.

The biological properties of *P. incarnata* and *S. rebaudiana* suggest that these botanical components may influence different aspects of the physiological response to stress. *P. incarnata* has been investigated particularly in relation to stress-associated and neuroactive effects, whereas *S. rebaudiana* has been investigated in poultry for its potential effects on immune responses, intestinal health, oxidative status, and meat quality. However, evidence regarding the combined use of these two botanical components in broilers, particularly during the period immediately before slaughter, remains limited. More specifically, it remains unclear whether a short-term phytogenic intervention administered exclusively during the final days before slaughter can produce measurable changes in endocrine, oxidative, and inflammatory biomarkers under commercial production conditions.

The question is particularly relevant because acute physiological responses can develop over a much shorter time frame than changes in growth, tissue deposition, or established skeletal morphology. A targeted five-day intervention therefore addresses a different biological question from conventional phytogenic feeding trials: whether nutritional modulation immediately before slaughter is associated with measurable changes in stress-related, oxidative, and inflammatory responses. Such an approach also minimizes the potential confounding effects of prolonged supplementation on growth performance and cumulative tissue development. Accordingly, the present study was designed to evaluate a phytogenic feed additive containing *P. incarnata* and *S. rebaudiana* extracts (PSPA) administered during the final five days before slaughter in broilers maintained under commercial production conditions.

In addition to endocrine, oxidative, and inflammatory responses, carcass characteristics and carcass quality were evaluated because physiological conditions during the preslaughter period may be reflected in commercially relevant processing outcomes. Previous experimental studies have demonstrated that preslaughter handling and transportation can influence physiological stress responses and postmortem meat quality characteristics [2,3]. Therefore, assessment of carcass weight, yield, and automated carcass classification alongside physiological biomarkers provides a broader evaluation of the potential consequences of a short-term preslaughter nutritional intervention.

Because bone morphology reflects cumulative processes of bone modelling and remodelling, bone morphology was additionally evaluated as an exploratory structural endpoint [24]. Although substantial alterations in established bone shape would not be expected within a five-day intervention, three-dimensional geometric morphometric analysis provides a sensitive approach for detecting subtle shape variation that may not be captured by conventional linear osteometric measurements [25]. This approach therefore allowed us to determine whether the physiological responses associated with short-term PSPA supplementation were accompanied by detectable changes in skeletal morphology.

To the best of our knowledge, no previous study has comprehensively evaluated the effects of a phytogenic feed additive containing *Passiflora incarnata* and *Stevia rebaudiana* extracts administered exclusively during the final five days before slaughter on endocrine, oxidative, and inflammatory biomarkers together with carcass characteristics, automated carcass quality classification, meat composition, and three-dimensional bone morphology in broilers under commercial production conditions. Therefore, the present study aimed to determine whether short-term PSPA supplementation during the final five days before slaughter was associated with measurable changes in endocrine, oxidative, and inflammatory biomarkers, carcass characteristics, meat composition, and three-dimensional bone morphology.

## 2. Materials and Methods

### 2.1. Experimental Design and Birds

A total of 106,240 one-day-old mixed-sex Ross 308 broiler chicks obtained from a commercial hatchery were used in this study. The experiment was conducted under commercial production conditions using a completely randomized design. Six commercial broiler houses (12 × 65 m) located on the same farm and operated during the same production cycle were included in the study. Until 37 days of age, all birds received the same commercial diets formulated according to the Ross 308 feeding programme [26]. At 38 days of age, the six houses were randomly assigned to one of two dietary treatments, with three houses allocated to the control group and three houses to the PSPA group. Each house was considered one experimental replicate and the experimental unit for treatment allocation. Birds in the control group (54,400 birds) continued to receive the commercial finisher diet, whereas birds in the PSPA group (51,840 birds) received the same commercial finisher diet supplemented with a phytogenic feed additive containing extracts of *P. incarnata* and *S. rebaudiana* Bertoni (PSPA; Nutrinexus S.L., Barcelona, Spain) during the last five days before slaughter (days 38–42). The additive was incorporated into the finisher diet at an inclusion rate of 1 kg/t during commercial feed manufacture.

All experimental diets were manufactured in the commercial feed mill supplying the farm. The commercial basal finisher diet consisted of 53.9% maize, 21.9% soybean meal, 11% full-fat soya, 7.9% wheat middlings, 2.9% blended oil, 0.6% limestone, 0.3% salt, and 1.5% other ingredients (including vitamin and mineral premixes, synthetic amino acids, and other micro-ingredients routinely used by the feed manufacturer).

All birds were managed according to the standard commercial management practices of the farm and the recommendations of the Ross 308 Broiler Management Handbook [27]. Feed and water were provided ad libitum throughout the study. Environmental conditions, lighting programme, ventilation, litter management, vaccination schedule, and health management were identical for all houses. As part of the routine commercial management programme, approximately 22% of the birds were removed from all houses at 34 days of age (thinning). This procedure was performed uniformly across all experimental houses before the initiation of dietary treatments.

All experimental procedures involving animals were approved by the Animal Experiments Local Ethics Committee of Ankara University (Approval No: 2025-14-161; Approval date: 23 July 2025) and were conducted in accordance with institutional guidelines for the care and use of experimental animals.

### 2.2. Chemical Analysis of Diets and Phytogenic Additive

Representative feed samples were collected from each experimental diet during commercial feed manufacture. One composite sample was prepared separately for the control and PSPA diets and analyzed in duplicate for dry matter, crude protein, ether extract, and crude ash according to the standard procedures of the Association of Official Analytical Chemists (AOAC) [28]. For the analysis of calcium and total phosphorus, diet samples were subjected to wet digestion prior to inductively coupled plasma-mass spectrometry (ICP-MS) measurement, following established procedures for animal feed analysis [29] as reported by [30]. The metabolizable energy content of the diet was calculated using Carpenter and Clegg’s equation as reported by Yalçın et al. [31]. The analyzed composition of finisher diets was given in Table 1.

The phytogenic feed additive was characterized by determining its total polyphenol content, individual phenolic compounds, and volatile compound profile. Total polyphenol content was determined according to the spectrophotometric method described by Hrncirik and Fritsche [32]. Briefly, homogenized samples were extracted with an 80:20 (*v*/*v*) methanol:water solution. Following extraction, the extracts were reacted with Folin–Ciocalteu reagent under alkaline conditions, and absorbance was measured at 725 nm using a UV-visible spectrophotometer (T-80+, PG Instruments, Leicestershire, UK). Quantification was performed by external calibration using gallic acid as the reference standard, and the results were expressed as mg gallic acid equivalents (GAE)/kg of additive. Individual phenolic compounds were analyzed by high-performance liquid chromatography with ultraviolet detection (HPLC-UV), using an analytical procedure adapted from the International Olive Oil Council (IOC) method [33]. Homogenized samples were extracted with 80:20 (*v*/*v*) methanol:water, filtered through membrane filters, and injected into an Agilent 1100 Series HPLC system equipped with a UV detector (Agilent Technologies, Santa Clara, CA, USA). Chromatographic separation was performed on an ACE 5 C18 column maintained at 25 °C using a mobile phase consisting of 0.2% phosphoric acid in water (solvent A) and methanol:acetonitrile (1:1, *v*/*v*) (solvent B) under a gradient elution program. The flow rate was 1 mL/min, the injection volume was 20 µL, and chromatograms were monitored at 280 nm. Individual phenolic compounds were identified and quantified by external calibration using authentic reference standards. The volatile compound profile was determined by gas chromatography-mass spectrometry (GC-MS). Volatile oil was first isolated from the phytogenic additive by hydrodistillation using a Clevenger apparatus, collected in xylene, and subsequently analyzed using an Agilent 5975 GC-MS system (Agilent Technologies, Santa Clara, CA, USA) equipped with an HP-5MS capillary column (30 m × 0.25 mm i.d., 0.25 µm film thickness). Helium was used as the carrier gas, and chromatographic separation was performed under a programmed oven temperature gradient at a constant flow rate. Volatile compounds were identified by comparing their mass spectra with those contained in the Flavor2, HPCH1607, and W8N05ST spectral libraries, together with retention index data when available.

### 2.3. Slaughter Procedure and Sample Collection

At 43 days of age, blood samples were collected from five male and five female broilers randomly selected from each of the three houses per group (10 birds per house; 30 birds per group). Birds were randomly caught from different locations within each house to minimize sampling bias. Approximately 3 mL of blood was collected from the brachial vein of each bird into serum collection tubes. The blood samples were allowed to clot at room temperature for 30 min and centrifuged at 1500× *g* for 10 min to obtain serum. Serum samples were stored at −40 °C until analysis of inflammatory cytokines (IL-1β, IL-6, TNF-α, IL-10), corticosterone, and oxidative stress biomarkers (TAS, TOS, and OSI).

At 43 days of age, all birds were slaughtered under standard commercial processing conditions in the same commercial slaughterhouse. Following evisceration, carcasses were chilled in an air-chilling system for 160 min until the internal carcass temperature reached 4 °C, while the air temperature was maintained between 1 and 1.5 °C. Carcass quality of all slaughtered birds was subsequently evaluated using the automated commercial vision grading system.

For carcass yield, tissue composition, and bone analyses, 50 broilers (25 males and 25 females) were randomly selected from each experimental house (150 birds per treatment). The 10 birds previously sampled for blood collection were included within this group of 50 birds. Individual pre-slaughter live weight and chilled carcass weight were recorded, after which samples of the right pectoralis major muscle, liver, and the right humerus, radius, ulna, carpometacarpus, femur, tibiotarsus, and tarsometatarsus were collected for subsequent chemical, biochemical, and geometric morphometric analyses.

### 2.4. Carcass Evaluation

For each individual bird selected for laboratory analyses, carcass yield (%) was calculated using its chilled carcass weight and corresponding pre-slaughter live weight according to the following equation:

Carcass yield (%) = (chilled carcass weight/pre-slaughter live weight) × 100

Commercial carcass quality was evaluated for all slaughtered broilers using the IRIS (The Intelligent Reporting, Inspection and Selection, JBT Marel, Boxmeer, The Netherlands) automated vision grading system installed at the commercial processing plant. After chilling, each carcass was automatically inspected and classified to the commercial quality standards routinely used by the processing plant. The proportions of A-grade carcasses, broken wings, breast defects, thigh defects, bloody wings, and other carcass defects were recorded separately for each experimental house. These house-level percentages were used as the experimental observations for statistical analysis.

### 2.5. Determination of Oxidative Stress Biomarkers

Serum total antioxidant status (TAS) and total oxidant status (TOS) were determined using commercially available colorimetric assay kits (Rel Assay Diagnostics, Gaziantep, Türkiye; TAS: Cat. No. RL0017, TOS: Cat. No. RL0024) according to the manufacturer’s instructions. TAS was expressed as mmol Trolox equivalent/L, whereas TOS was expressed as μmol H_2_O_2_ equivalent/L. For calculation of the oxidative stress index (OSI), TOS values were first converted from μmol H_2_O_2_ equivalent/L to mmol H_2_O_2_ equivalent/L. OSI was then calculated using the following equation:

OSI = (TOS (mmol H_2_O_2_ equivalent/L)/TAS (mmol Trolox equivalent/L) × 100,

as previously described by Onbaşılar et al. [34].

### 2.6. Determination of Inflammatory Cytokines and Corticosterone

Serum concentrations of interleukin-1 beta (IL-1β), interleukin-6 (IL-6), tumor necrosis factor-alpha (TNF-α), interleukin-10 (IL-10), and corticosterone (CORT) were determined using commercially available chicken-specific enzyme-linked immunosorbent assay (ELISA) kits (BT LAB Bioassay Technology Laboratory, Shanghai, China) according to the manufacturer’s instructions. The kits used were IL-1β (Cat. No. EA0003Ch), IL-6 (Cat. No. E0004Ch), TNF-α (Cat. No. EA0010Ch), IL-10 (Cat. No. EA0015Ch), and corticosterone (Cat. No. EA0019Ch). Serum samples and standards were analyzed in duplicate. Absorbance was measured using a microplate reader at the wavelength recommended by the manufacturer, and analyte concentrations were calculated from standard curves generated for each assay. Cytokine concentrations were expressed as pg/mL, whereas corticosterone concentrations were expressed as ng/mL.

### 2.7. Chemical Composition of Breast Muscle and Liver

Samples of the right pectoralis major muscle and liver collected from each bird were first freeze-dried using a laboratory lyophilizer and then finely ground to obtain homogeneous samples. The lyophilized samples were analyzed for dry matter, crude protein (Kjeldahl, N × 6.25), crude fat (ether extract), and crude ash according to the standard procedures of the AOAC [28]. Each sample was analyzed in duplicate, and the mean of the two determinations was used as the value for each bird in the statistical analysis. Individual birds constituted the observational units for laboratory measurements, whereas the house was considered the experimental unit because dietary treatments were applied at the house level.

### 2.8. Bone Sampling

Following carcass evaluation, the right humerus, radius, ulna, carpometacarpus, femur, tibiotarsus, and tarsometatarsus were carefully excised from each bird. Adhering muscles, tendons, ligaments, and other soft tissues were manually removed using scalpels and forceps without damaging the bone surface. Each bone was individually labelled with a unique identification code corresponding to the treatment group, experimental house, sex, and bird number. The cleaned bones were stored in sealed polyethylene bags and kept at 4 °C until transportation to the imaging laboratory, where CT scanning and three-dimensional geometric morphometric analyses were performed.

### 2.9. Computed Tomography (CT) Scanning and Three-Dimensional Reconstruction

Computed tomography images of the right humerus, radius, ulna, carpometacarpus, femur, tibiotarsus, and tarsometatarsus were acquired using a 256-slice CT scanner (SOMATOM Definition Flash, Siemens Healthineers, Forchheim, Germany). All bones were scanned under identical acquisition conditions using a tube voltage of 120 kV, a tube current of 251 mA, a 512 × 512 image matrix, and 0.63-mm slice thickness.

The acquired Digital Imaging and Communications in Medicine (DICOM) image files were imported into 3D Slicer (version 5.8.1) for image processing and three-dimensional reconstruction [35]. Bone segmentation was performed using a semi-automatic threshold-based procedure. To ensure methodological consistency, all specimens were segmented by the same investigator using identical software settings. A fixed threshold range of 210.58–2720.00 was applied to all CT datasets. Following segmentation, three-dimensional surface models were reconstructed from the axial image series and visually inspected to verify segmentation quality and anatomical completeness before landmark digitization and subsequent geometric morphometric analyses [34,35].

### 2.10. Landmark Acquisition

Three-dimensional anatomical landmarks were digitized on each reconstructed bone model using 3D Slicer (version 5.8.1) [36]. Landmark configurations were established separately for the humerus, radius, ulna, carpometacarpus, femur, tibiotarsus, and tarsometatarsus. Landmarks consisted of a combination of type I, type II, and type III (semi-landmarks) according to the classification proposed by Bookstein [37]. Type I landmarks were positioned on anatomically discrete homologous structures, type II landmarks were defined by local geometric features, whereas semi-landmarks were distributed along anatomically homologous curves and surfaces to characterize continuous shape variation. Landmark definitions and anatomical locations were established based on previous osteological and geometric morphometric studies [30,38,39]. The complete landmark configuration and anatomical descriptions for each bone are presented in Table 2. All landmarks were digitized by the same investigator using standardized anatomical criteria to ensure consistency among specimens.

The complete landmarking procedure was repeated independently by the same observer for all specimens, and the two landmark datasets were subsequently compared. To determine whether the repeated digitization processes resulted in statistically significant differences in shape, the repeated landmark configurations were compared using multivariate analysis of variance (MANOVA). No statistically significant differences were detected between the repeated landmark digitizations (*p* > 0.05 for all bones), indicating that landmark digitization by the same observer was highly consistent across repeated measurements. Accordingly, the first landmark dataset was retained for all subsequent geometric morphometric analyses.

### 2.11. Geometric Morphometric Analysis

Geometric morphometric analyses were performed separately for the humerus, radius, ulna, carpometacarpus, femur, tibiotarsus, and tarsometatarsus using PAST software (version 4.03) [40]. Landmark configurations obtained from each bone were first subjected to Generalized Procrustes Analysis (GPA) to remove variation attributable to specimen position, orientation, and scale while preserving biologically meaningful shape information [36]. Following GPA, principal component analysis (PCA) was performed independently for each bone using the Procrustes-aligned landmark coordinates. All principal component (PC) scores generated by the PCA were retained for subsequent multivariate statistical analyses; therefore, no subjective selection criterion based on explained variance or biological interpretation was applied.

Overall shape differences between treatment groups (Control vs. PSPA) and between sexes (Female vs. Male) were evaluated separately for each bone using MANOVA based on the complete set of PC scores obtained from the Procrustes Analysis. Additional MANOVA was performed to compare the four experimental groups (Control Female, Control Male, PSPA Female, and PSPA Male). To control for multiple testing across the bone-specific analyses, *p*-values from the overall MANOVA tests were adjusted using the Benjamini–Hochberg false discovery rate (FDR) procedure. When an overall MANOVA was significant, pairwise multivariate comparisons were subsequently performed to identify the groups responsible for the observed shape differences. *p*-values from these pairwise comparisons were also adjusted using the Benjamini–Hochberg FDR procedure, and statistical significance was accepted at FDR-adjusted *p* < 0.05.

To facilitate biological interpretation of shape variation, reconstructed three-dimensional bone models corresponding to the positive and negative extremes of the principal component axes were generated and superimposed. Morphological changes were further visualized using lollipop plots, in which displacement vectors illustrate both the direction and magnitude of landmark movement. These visualizations enabled identification of the anatomical regions contributing most prominently to shape variation in the humerus, radius, ulna, carpometacarpus, femur, tibiotarsus, and tarsometatarsus.

### 2.12. Statistical Analysis

Statistical analyses were performed using IBM SPSS Statistics for Windows, Version 22.0 (IBM Corp., Armonk, NY, USA) unless otherwise stated. Data are presented according to the statistical method used for each analysis. Results from mixed-effects models are reported as estimated marginal means (least-squares means, LS means) ± standard error (SE), whereas carcass quality data are presented as house-level unweighted means ± standard deviation (SD) with ranges. Geometric LS means with 95% confidence intervals (CI) are presented for log-transformed variables. Statistical significance was accepted at *p* < 0.05. Because dietary treatments were applied at the house level, the house was considered the experimental unit for treatment allocation, whereas individual birds constituted the observational units for laboratory measurements. In all factorial analyses, treatment (Control vs. PSPA), sex (male vs. female), and their interaction were included as fixed effects. Significant interactions were further explored using Bonferroni-adjusted pairwise comparisons.

Carcass weight and carcass yield: Individual carcass weight and carcass yield were analyzed using linear mixed-effects models (LMMs) fitted by restricted maximum likelihood (REML). Treatment, sex, and their interaction were included as fixed effects, whereas house was included as a random intercept to account for clustering of birds within houses. Pre-slaughter live weight was included as a covariate in the carcass weight but not in the carcass yield because live weight is incorporated into the calculation of carcass yield.

Carcass quality traits: Carcass quality characteristics were analyzed at the house level, with the house considered the independent experimental unit. For each carcass quality trait, the percentage of carcasses in the corresponding category was calculated separately for each house. House-level percentages were summarized as the unweighted mean ± standard deviation (SD) and range across the three independent houses in each treatment group. Between-group differences were expressed in percentage points and calculated as the mean house-level percentage in the PSPA group minus that in the control group. Given the small number of independent experimental units (three houses per treatment), between-group comparisons were performed using two-sided exact permutation tests at the house level, with treatment labels permuted across the six independent houses. Exact *p*-values were calculated from the complete permutation distribution.

Serum biochemical variables and tissue composition: Serum inflammatory cytokines (IL-1β, IL-6, TNF-α, and IL-10), corticosterone, oxidative stress biomarkers (TAS, TOS, and OSI), and the composition of breast muscle and liver (dry matter, crude protein, crude fat, and ash) were analyzed using linear mixed-effects models fitted by restricted maximum likelihood (REML). Treatment (Control vs. PSPA), sex (male vs. female), and their interaction were included as fixed effects, whereas house was included as a random intercept to account for clustering of birds within houses. Type III tests of fixed effects were used for statistical inference. IL-1β, IL-6, and TNF-α were analyzed after log10 transformation and are presented as back-transformed geometric LS means with 95% confidence intervals (CI). All remaining variables are presented on their original measurement scale.

Geometric morphometric analysis: Geometric morphometric analyses were performed separately for the humerus, radius, ulna, carpometacarpus, femur, tibiotarsus, and tarsometatarsus using PAST software (Version 4.03) [40]. Landmark configurations were first aligned using Generalized Procrustes Analysis (GPA), followed by PCA of the Procrustes coordinates. All PC scores generated by PCA were retained for subsequent statistical analyses. Therefore, no PCs were excluded based on explained variance or subjective biological interpretation. Differences in bone shape between treatment groups and sexes, as well as among the four experimental groups (Control Female, Control Male, PSPA Female, and PSPA Male), were evaluated using MANOVA based on the complete set of PC scores. When significant overall differences were detected, pairwise multivariate comparisons were performed to identify the groups responsible for the observed shape variation. To control for multiple testing, FDR correction was applied to both the overall MANOVA tests and the subsequent pairwise comparisons. No formal factorial MANOVA including a treatment × sex interaction term was performed because comparisons were based on separate two-group (treatment and sex) analyses and four-group multivariate comparisons. Statistical significance was accepted at an FDR-adjusted *p* < 0.05.

## 3. Results

### 3.1. Phytochemical Characterization of the Phytogenic Feed Additive

The chemical composition of the phytogenic feed additive (PSPA) is presented in Table 3 and Table 4. Total polyphenol content was 5841 mg/kg. Among the HPLC-quantified individual phenolic compounds, caffeic acid (9.92 mg/kg) was the most abundant, followed by vanillin, apigenin, vanillic acid, *p*-coumaric acid, and luteolin. Analysis of volatile compounds identified a complex phytochemical profile comprising aldehydes, terpenes, sesquiterpenes, phenolic compounds, and oxygenated terpenoids. The most abundant volatile constituent was *m*-tolualdehyde (34.7%), followed by 2-methylbenzaldehyde (9.9%), spathulenol (7.6%), ambrettolide (6.1%), acetophenone (4.1%), 2-methoxy-4-vinylphenol (2.5%), and phytol (2.2%). Several other biologically active compounds were detected in lower proportions, including thymoquinone, carvacrol, eugenol, limonene, linalool, valencene, and caryophyllene oxide. BHA was also detected during GC-MS analysis. However, because the formulation of the commercial additive is proprietary, it cannot be determined whether this compound originated from the additive formulation itself or was present as an antioxidant used during commercial manufacturing.

### 3.2. Carcass Yield and Carcass Quality

The effects of dietary PSPA supplementation on carcass weight, carcass yield, and carcass quality are presented in Table 5 and Table 6.

After adjustment for pre-slaughter live weight, carcass weight was significantly higher in the treatment group than in the control group (*p* = 0.002). Male birds exhibited greater carcass weights than females (*p* < 0.001), whereas no significant Group × Sex interaction was detected. Carcass yield was not affected by dietary treatment (*p* = 0.180); however, males showed a higher carcass yield than females (*p* < 0.001). Carcass quality outcomes showed small numerical differences between treatments, although none reached statistical significance in the house-level exact permutation analyses. The mean proportion of A-grade carcasses was numerically higher in the PSPA group than in the control group (66.43% vs. 62.62%), corresponding to a difference of +3.82 percentage points. Smaller numerical differences were observed for thigh defects, breast defects, bloody wings, and other carcass defects, with no statistically significant between-group differences.

### 3.3. Breast Meat and Liver Composition

The effects of dietary PSPA supplementation and sex on the proximate composition of breast meat and liver are presented in Table 7. Dietary PSPA supplementation did not significantly affect the dry matter, crude protein, crude fat, or ash contents of either breast meat or liver (*p* > 0.05). Likewise, no significant effects of sex or treatment x sex interaction were observed for any compositional variable (*p* > 0.05), indicating that the proximate composition of breast muscle and liver was not influenced by PSPA supplementation during the final five days before slaughter.

### 3.4. Serum Inflammatory Cytokines and Oxidative Stress Biomarkers

The effects of dietary PSPA supplementation and sex on serum inflammatory cytokines, corticosterone, and oxidative stress biomarkers are presented in Table 8. Dietary supplementation with PSPA resulted in significantly lower serum concentrations of the pro-inflammatory cytokines IL-1β, IL-6, TNF-α, as well as corticosterone, TOS, and OSI (*p* < 0.05). In contrast, the anti-inflammatory cytokine IL-10 was significantly increased in birds receiving PSPA compared with the control group (*p* = 0.048). TAS was not affected by dietary supplementation (*p* > 0.05). No significant effects of sex or Group × Sex interactions were detected for any of the measured variables (*p* > 0.05).

### 3.5. Three-Dimensional Geometric Morphometrics

Three-dimensional geometric morphometric analyses revealed relatively limited treatment-related differences in overall bone shape (Table 9, Table 10 and Table 11; Figure 1, Figure 2, Figure 3 and Figure 4). When groups were compared irrespective of sex, a nominally significant difference was detected only for the femur (*p* = 0.043), whereas the remaining bones were not affected by treatment (Table 9). Because treatment was applied only during the final five days before slaughter, this isolated finding should be interpreted cautiously. In contrast, significant sex-related differences were observed for the ulna (*p* = 0.019), carpometacarpus (*p* = 0.036), and tibiotarsus (*p* = 0.0006). Exploratory four-group comparisons revealed significant pairwise differences involving the PSPA female group for the carpometacarpus and tibiotarsus, whereas the ulna and tibiotarsus differed only between PSPA females and PSPA males.

The PCA scatter plots showed substantial overlap among treatment groups for most bones, with only limited visual separation for the femur. The carpometacarpus and tibiotarsus primarily reflected sex-related variation rather than treatment-related differences (Figure 1, Figure 2, Figure 3 and Figure 4).

## 4. Discussion

The present study evaluated the effects of PSPA, a phytogenic supplement containing *P. incarnata* and *S. rebaudiana*, administered during the final five days before slaughter. This supplementation period immediately preceded commercial preslaughter procedures, during which birds were exposed to acute stressors such as feed withdrawal, catching, loading, transportation, and preslaughter handling, which activate the HPA axis, increase corticosterone secretion, and contribute to oxidative and inflammatory responses. These physiological disturbances may affect animal welfare, carcass quality, and meat characteristics. Previous studies have largely focused on the effects of phytogenic feed additives administered throughout the entire growing period [41,42]. In contrast, the present study evaluated supplementation restricted to the final five days before slaughter. Under these conditions, PSPA supplementation was associated with significant changes in endocrine, oxidative, and inflammatory biomarkers, suggesting that short-term phytogenic supplementation may influence acute physiological responses during the pre-slaughter period.

Unlike many previous studies, the present work included a comprehensive phytochemical characterization of the tested additive, providing a biological framework for interpreting the observed physiological responses. Rather than attributing the effects of PSPA to a single bioactive constituent, the findings should be interpreted in the context of its complex phytochemical composition. Phytogenic feed additives are generally considered to exert their biological activity through the complementary actions of multiple phenolic compounds, flavonoids, terpenoids, and other secondary plant metabolites acting on several physiological pathways simultaneously [7,43,44]. Accordingly, the endocrine, oxidative, and inflammatory responses observed in the present study are biologically consistent with the overall phytochemical profile of PSPA, although the relative contribution of individual constituents cannot be determined.

Among the HPLC-quantified phenolic compounds, caffeic acid and *p*-coumaric acid are recognized hydroxycinnamic acids with strong free radical-scavenging activity, whereas luteolin and apigenin have been reported to suppress pro-inflammatory cytokine production while enhancing endogenous antioxidant defenses through regulation of redox-sensitive signalling pathways [45,46]. Apigenin, a major flavonoid of *P. incarnata*, has additionally been shown to interact with benzodiazepine-binding sites of GABA_A_ receptors, contributing to the anxiolytic properties traditionally attributed to passionflower [18,22]. The volatile fraction may further contribute to the biological activity of PSPA because it contains numerous bioactive terpenes and terpenoids. Many of these compounds have individually been reported to exhibit antioxidant and anti-inflammatory activities, whereas some have also been associated with immunomodulatory or neuroactive effects, potentially complementing the actions of the phenolic constituents [47]. Collectively, the identified constituents provide a plausible phytochemical basis that may contribute to the antioxidant, anti-inflammatory, and stress-related responses observed following PSPA supplementation.

The apparent discrepancy between the total (poly)phenol content determined by the Folin–Ciocalteu assay and the sum of the individually quantified phenolic compounds by HPLC should be interpreted in light of the different analytical principles of these methods. The Folin–Ciocalteu assay provides an overall estimate of total polyphenol content based on the reducing capacity of the sample, whereas HPLC enables the identification and quantification of specific compounds for which appropriate reference standards are available. Therefore, the value obtained by the Folin–Ciocalteu assay should not be expected to correspond to the arithmetic sum of the individually quantified HPLC compounds. In addition, the Folin–Ciocalteu reagent may react with other reducing substances present in complex plant matrices, which can influence the measured value [48].

Previous studies provide biological plausibility for potential effects of *P. incarnata* on stress-related responses and *S. rebaudiana* on antioxidant, inflammatory, and intestinal processes. Supporting this biological plausibility, *P. incarnata* supplementation reduced aggressive behaviour, ear lesions, and salivary cortisol concentrations in weaned pigs [23], whereas dietary stevia supplementation has been associated with reduced inflammatory responses [12], enhanced humoral immunity [13], beneficial modulation of the cecal microbiota [14], reduced lipid peroxidation under heat-stress conditions [15], and increased antioxidant enzyme activities together with improved meat quality [16] in broilers and quails. These findings provide a plausible biological basis for the responses observed following PSPA supplementation in the present study and suggest that short-term supplementation may warrant further evaluation as a practical preslaughter nutritional strategy. However, because the two botanical components were not evaluated separately, their individual contributions or any additive or synergistic effects cannot be determined.

Although growth performance was not evaluated because PSPA was administered only during the final five days before slaughter, carcass yield and carcass quality provide practical outcomes relevant to commercial processing. The preslaughter period involves several acute stressors that may influence physiological responses and carcass characteristics. Therefore, evaluating carcass traits alongside endocrine and inflammatory biomarkers provides a practical perspective on whether changes in carcass characteristics accompany the physiological responses associated with short-term PSPA supplementation.

In the present study, supplementation with PSPA significantly increased carcass weight after adjustment for preslaughter live weight, whereas carcass yield remained unchanged. Although none of the carcass quality variables reached statistical significance, numerical trends consistently favored PSPA supplementation. Broilers receiving PSPA exhibited a higher proportion of Grade A carcasses (66.43% vs. 62.62%) together with lower incidences of thigh, breast, and other carcass defects. Collectively, these findings indicate numerical differences favouring PSPA supplementation, but do not provide statistical evidence of improved carcass quality.

The absence of statistically significant differences in carcass grading should nevertheless be interpreted cautiously. Carcass defects are multifactorial traits influenced not only by physiological stress but also by catching procedures, transportation conditions, stocking density, slaughterhouse handling, processing equipment, and random mechanical injuries. Furthermore, although more than 100,000 broilers were included in the commercial trial, carcass quality was statistically evaluated using only three independent houses per treatment. Consequently, the experimental unit was the house rather than the individual broiler, resulting in a limited number of independent experimental units for statistical inference and reduced precision of the estimates. Thus, the observed differences remain compatible with both small beneficial effects and no treatment effect.

From a commercial perspective, even modest changes in carcass classification could potentially be relevant because carcass grading may influence product value and downgrading. However, this potential practical relevance should be confirmed in a large number of independent commercial houses.

The reduction in serum corticosterone indicates an altered endocrine stress response during the preslaughter period. Corticosterone is the principal glucocorticoid hormone in birds and a widely used indicator of stress-related HPA axis activation [4,49]. The significantly lower corticosterone concentrations observed in PSPA-supplemented birds therefore suggest that short-term PSPA supplementation was associated with attenuation of the endocrine stress response during the final five days before slaughter. However, the relationship between this endocrine response and the numerical differences observed in carcass-quality traits cannot be established from the present data.

Neither breast muscle nor liver proximate composition was affected by PSPA supplementation. In contrast, longer-term supplementation with *S. rebaudiana* extract has been reported to improve several meat quality characteristics, including pH, color, tenderness, and crude fat content in broilers [17]. Therefore, the absence of compositional changes in the present study may partly reflect the brief five-day supplementation period rather than an absence of biological effects. In contrast, physiological responses such as changes in corticosterone, oxidative stress, and inflammatory biomarkers can occur more rapidly than changes in tissue chemical composition, which generally reflect longer-term nutrient deposition. These findings suggest that the detectable responses to short-term PSPA supplementation were primarily observed in circulating physiological biomarkers rather than in the proximate composition of muscle and liver tissues. Because pre-slaughter stress may influence postmortem meat quality, future studies should also evaluate technological meat quality traits, including pH, color, drip loss, cooking loss, water-holding capacity, shear force, and oxidative stability, to determine whether the physiological responses associated with PSPA supplementation are accompanied by changes in postmortem meat quality.

PSPA supplementation was associated with significantly reduced serum TOS and OSI, whereas TAS remained unchanged. These findings indicate that PSPA primarily reduced systemic oxidant burden rather than increasing the overall circulating antioxidant capacity. Oxidative stress develops when reactive oxygen species production exceeds the capacity of endogenous antioxidant defense systems, resulting in oxidative damage to lipids, proteins, and nucleic acids and ultimately impairing cellular function [5]. In commercial broiler production, preslaughter procedures such as feed withdrawal, catching, loading, transportation, and handling are well-recognized stressors capable of disturbing redox homeostasis. Therefore, reducing oxidative stress immediately before slaughter may help preserve redox balance, support animal welfare, and contribute to improved post-slaughter product quality [5].

The reduction in oxidative status observed in the present study is biologically consistent with the phytochemical profile of PSPA, which contains multiple phenolic compounds and volatile constituents with documented antioxidant properties. Rather than acting through a single dominant constituent, phytogenic feed additives are generally considered to exert their biological activity through complementary interactions among multiple bioactive compounds [7,43]. Accordingly, the reduced oxidative status may therefore be related to the combined antioxidant properties of phenolic and volatile constituents present in PSPA [50]. The absence of a significant increase in TAS should not be interpreted as evidence of a lack of antioxidant activity. Whereas TAS reflects the cumulative antioxidant capacity of plasma, TOS represents the systemic oxidant burden generated under stressful conditions. Consequently, the observed pattern may reflect a reduction in systemic oxidant burden without a measurable increase in circulating total antioxidant capacity. Because OSI integrates both oxidant and antioxidant status, the concurrent reductions in TOS and OSI indicate a more favourable systemic redox status despite the absence of significant changes in TAS [5].

These findings are consistent with previous poultry studies reporting antioxidant effects of stevia-containing phytogenic supplements. Bayril [15] observed reduced lipid peroxidation in heat-stressed quails receiving a combination of stevia and ginger extracts, whereas Ibrahim et al. [16] reported increased activities of antioxidant enzymes together with improved oxidative stability of breast meat in broilers supplemented with stevia. Unlike those studies, which administered stevia throughout the growing period, the present findings indicate that short-term supplementation was associated with favourable changes in oxidative-status biomarkers during the final five days before slaughter.

One of the principal findings of the present study was the attenuation of the inflammatory response during the preslaughter period. Birds receiving PSPA exhibited significantly lower serum concentrations of the pro-inflammatory cytokines IL-1β, IL-6, and TNF-α, together with higher IL-10 concentrations and reduced corticosterone levels. Collectively, these findings indicate that PSPA supplementation was associated with a less pro-inflammatory systemic cytokine profile rather than generalized suppression of immune function. The concurrent changes in endocrine, oxidative status, and inflammatory biomarkers are consistent with the established interplay between stress-related neuroendocrine responses and immune regulation, together with the role of redox balance in stress adaptation and homeostasis [5,51].

The immunomodulatory findings are also broadly consistent with previous studies of stevia supplementation in poultry. Daneshyar et al. [12] reported attenuation of the pro-inflammatory response following immune stimulation receiving dietary stevioside supplementation, whereas Jiang et al. [52] demonstrated protection against lipopolysaccharide-induced intestinal injury through attenuation of oxidative stress and inflammatory responses. Molina-Barrios et al. [13] further reported enhanced humoral immune responses, while Medeot et al. [14] observed favourable modulation of the cecal microbiota, including changes in the relative abundance of several bacterial genera and a reduction in *Escherichia-Shigella*, suggesting potential effects on intestinal microbial homeostasis. These findings provide biological plausibility for a contribution of the stevia component to the inflammatory changes observed in the present study. However, because PSPA contained both *P. incarnata* and *S. rebaudiana* extracts and the individual components were not tested separately, their respective contributions cannot be determined.

The observed changes should nevertheless be interpreted within the context of the short preslaughter intervention. Overall, PSPA supplementation was associated with favourable changes in measured endocrine, oxidative status, and inflammatory biomarkers, but these findings do not establish improved physiological resilience or causality. Further studies involving independent commercial houses, longer observation periods, and additional physiological and slaughter-related endpoints are warranted.

Three-dimensional geometric morphometric analysis was included to determine whether the marked endocrine, oxidative, and inflammatory changes observed during the preslaughter period were accompanied by detectable alterations in bone morphology. Although substantial changes in established bone morphology are not expected over a five-day intervention, CT-based geometric morphometrics provides a sensitive approach for detecting subtle shape variation that may not be captured by conventional linear osteometric measurements [25]. Bone morphology was therefore evaluated as an exploratory structural endpoint under commercial production conditions.

Under the conditions of the present study, PSPA supplementation provided limited evidence of treatment-related changes in bone morphology. Among the seven bones examined, the femur showed a significant treatment-related difference after FDR adjustment, whereas no significant treatment effects were detected for the remaining bones. Given the short supplementation period and the isolated nature of this finding, the femoral difference should be interpreted cautiously. In contrast, sex-related differences were more consistently observed, particularly for the ulna, carpometacarpus, and tibiotarsus, indicating that sex was an important source of variation in bone shape in these skeletal elements.

These findings are biologically plausible because bone morphology reflects the cumulative modelling and remodelling processes that generally develop over extended periods through sustained osteoblast and osteoclast activity. Accordingly, measurable morphological responses to nutritional interventions may be more likely to emerge following longer supplementation periods than after short-term treatments [24,30,34]. Thus, the absence of widespread bone-shape differences should be interpreted in the context of the five-day intervention period rather than as evidence against potential longer-term skeletal effects.

Interestingly, the limited bone response contrasted with the significant reductions in corticosterone, oxidative-status indices, and inflammatory biomarkers. Experimental studies have demonstrated that oxidative stress and prolonged glucocorticoid exposure can adversely affect bone remodelling by impairing osteoblast activity and promoting osteoclastogenesis [53,54,55]. Although the present experiment was not designed to evaluate bone metabolism directly, the observed biomarker profile provides a possible biological context in which longer-term effects on bone remodelling could be investigated. Whether such physiological changes ultimately translate into measurable improvements in bone morphology or mechanical strength following longer supplementation periods cannot be determined from the present study and remains to be established.

Future studies incorporating longer supplementation protocols together with complementary assessments including bone mineral density, biomechanical testing, histomorphometry, and bone turnover biomarkers will be valuable for determining whether the physiological effects observed during the preslaughter period are accompanied by detectable changes in skeletal quality.

## 5. Conclusions

The present study demonstrates that short-term supplementation with a phytogenic feed additive containing *Passiflora incarnata* and *Stevia rebaudiana* during the final five days before slaughter was associated with favorable changes in endocrine, oxidative, and inflammatory biomarkers in broilers under commercial production conditions. PSPA supplementation reduced serum corticosterone concentrations, decreased systemic oxidative stress as indicated by lower TOS and OSI, reduced the circulating concentrations of IL-1β, IL-6, and TNF-α, and increased IL-10, indicating a less pro-inflammatory and more favorable systemic physiological profile during the preslaughter period.

These physiological responses were accompanied by a significant increase in carcass weight after adjustment for preslaughter live weight and numerical differences favouring PSPA in carcass classification, although the latter did not reach statistical significance. In contrast, only limited changes were detected in bone morphology, with a significant treatment-related difference observed only for the femur after FDR adjustment. These findings suggest that the five-day supplementation period was associated with measurable physiological responses, whereas widespread alterations in established bone shape were not detected.

Overall, the present findings provide novel evidence that targeted phytogenic supplementation immediately before slaughter may influence endocrine, oxidative, and inflammatory responses in broiler chickens under commercial production conditions. Future studies involving a larger number of independent commercial houses, longer supplementation periods, and comprehensive postmortem meat-quality measurements are warranted to determine whether these physiological responses are accompanied by reproducible improvements in carcass and meat quality. Longer-term studies incorporating bone mineral density, biomechanical properties, histomorphometry, and bone turnover biomarkers are also needed to determine whether extended supplementation is associated with measurable changes in bone quality.

## Figures and Tables

**Figure 1:**
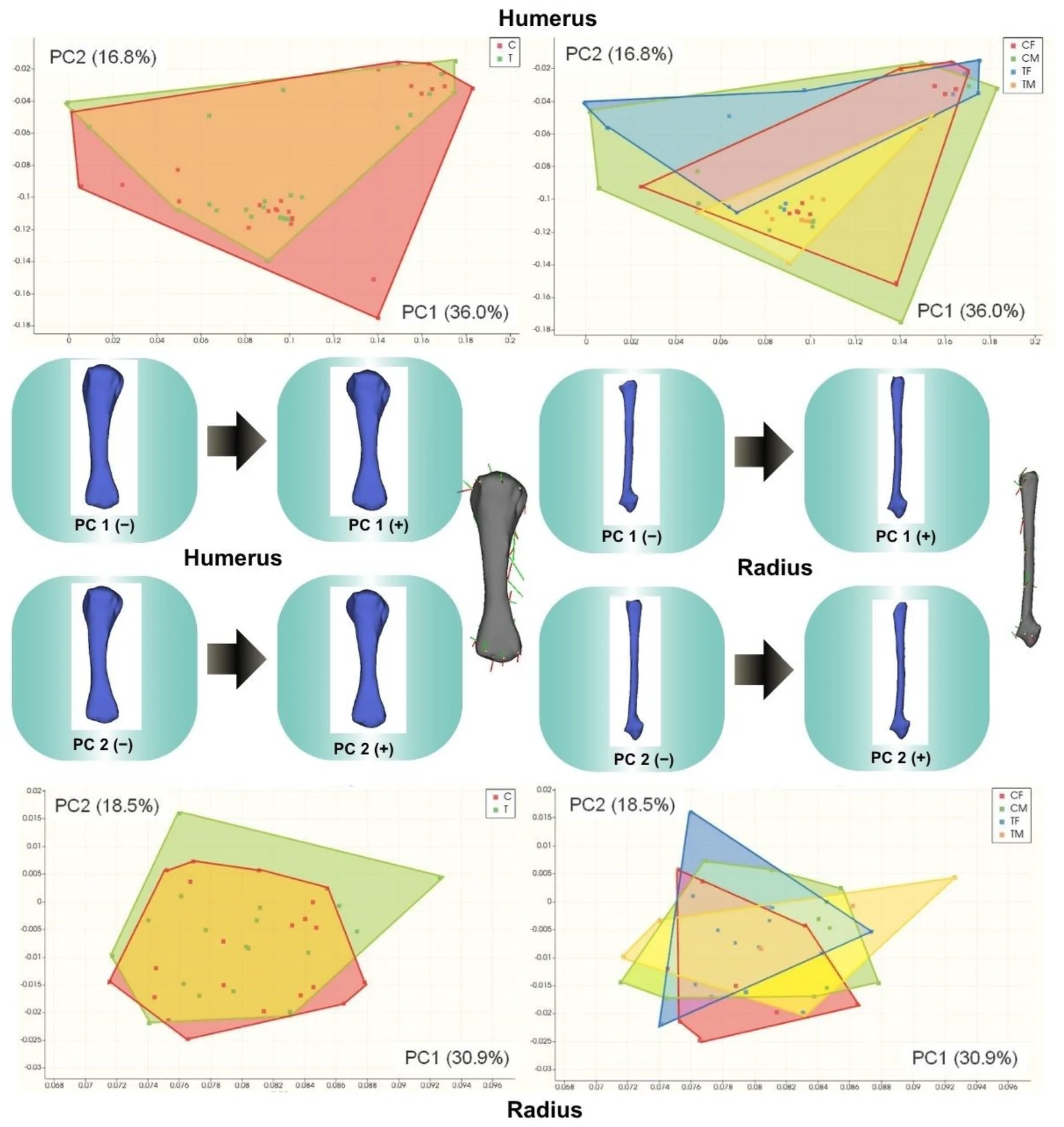
Principal component analysis (PCA) of humerus (**top**) and radius (**bottom**) shape variation obtained from three-dimensional geometric morphometric analysis. The left graphs at the top and bottom: Two-group comparisons showing the control (C, red) and treatment (T, green) groups for the respective bone. Right graphs at the top and bottom: Four-group subdivisions that separate the groups associated with the relevant bone by gender: control-female (CF, red), control-male (CM, green), treatment-female (TF, blue), and treatment-male (TM, yellow). The colored polygons represent the scatter plots of each group’s observations within the PCA plot. Shape changes associated with the positive direction of PC1 and PC2 are illustrated adjacent to each axis. Percentages indicate the proportion of total shape variation explained by each principal component.

**Figure 2:**
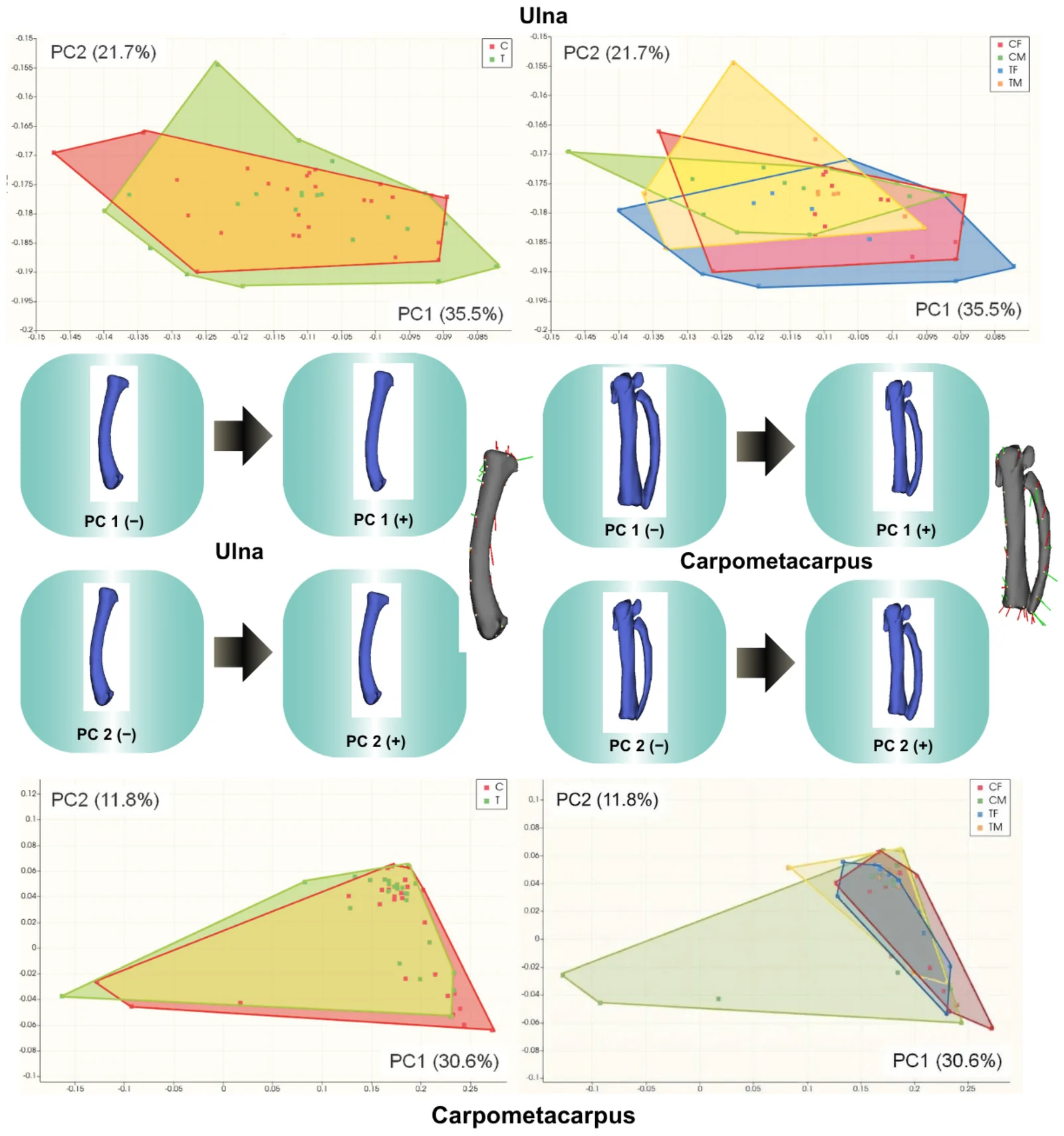
Principal component analysis (PCA) of ulna (**top**) and carpometacarpus (**bottom**) shape variation obtained from three-dimensional geometric morphometric analysis. The left graphs at the top and bottom: Two-group comparisons showing the control (C, red) and treatment (T, green) groups for the respective bone. Right graphs at the top and bottom: Four-group subdivisions that separate the groups associated with the relevant bone by gender: control-female (CF, red), control-male (CM, green), treatment-female (TF, blue), and treatment-male (TM, yellow). The colored polygons represent the scatter plots of each group’s observations within the PCA plot. Shape changes associated with the positive direction of PC1 and PC2 are illustrated adjacent to each axis. Percentages indicate the proportion of total shape variation explained by each principal component.

**Figure 3:**
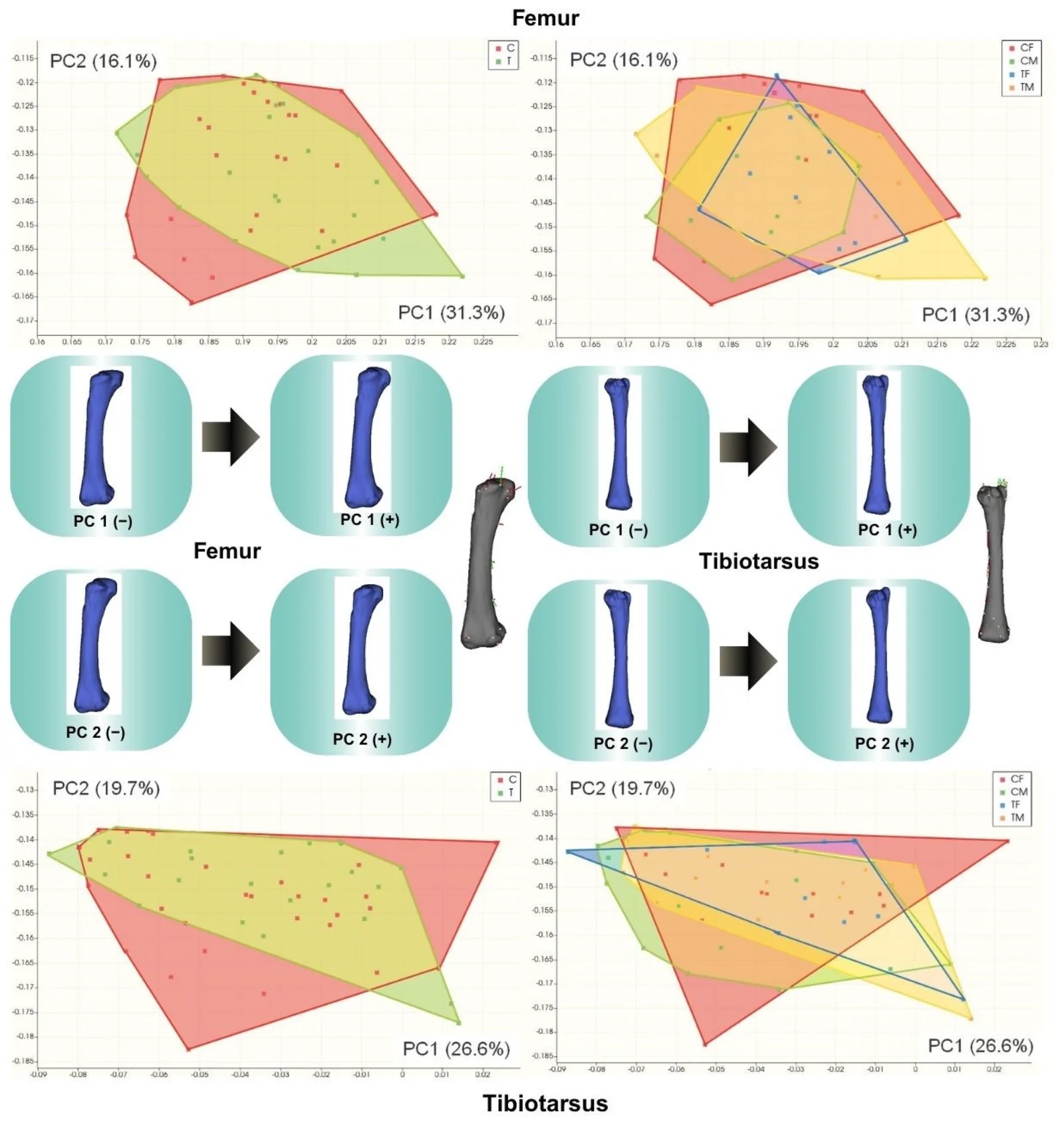
Principal component analysis (PCA) of femur (**top**) and tibiotarsus (**bottom**) shape variation obtained from three-dimensional geometric morphometric analysis. The left graphs at the top and bottom: Two-group comparisons showing the control (C, red) and treatment (T, green) groups for the respective bone. Right graphs at the top and bottom: Four-group subdivisions that separate the groups associated with the relevant bone by gender: control-female (CF, red), control-male (CM, green), treatment-female (TF, blue), and treatment-male (TM, yellow). The colored polygons represent the scatter plots of each group’s observations within the PCA plot. Shape changes associated with the positive direction of PC1 and PC2 are illustrated adjacent to each axis. Percentages indicate the proportion of total shape variation explained by each principal component.

**Figure 4:**
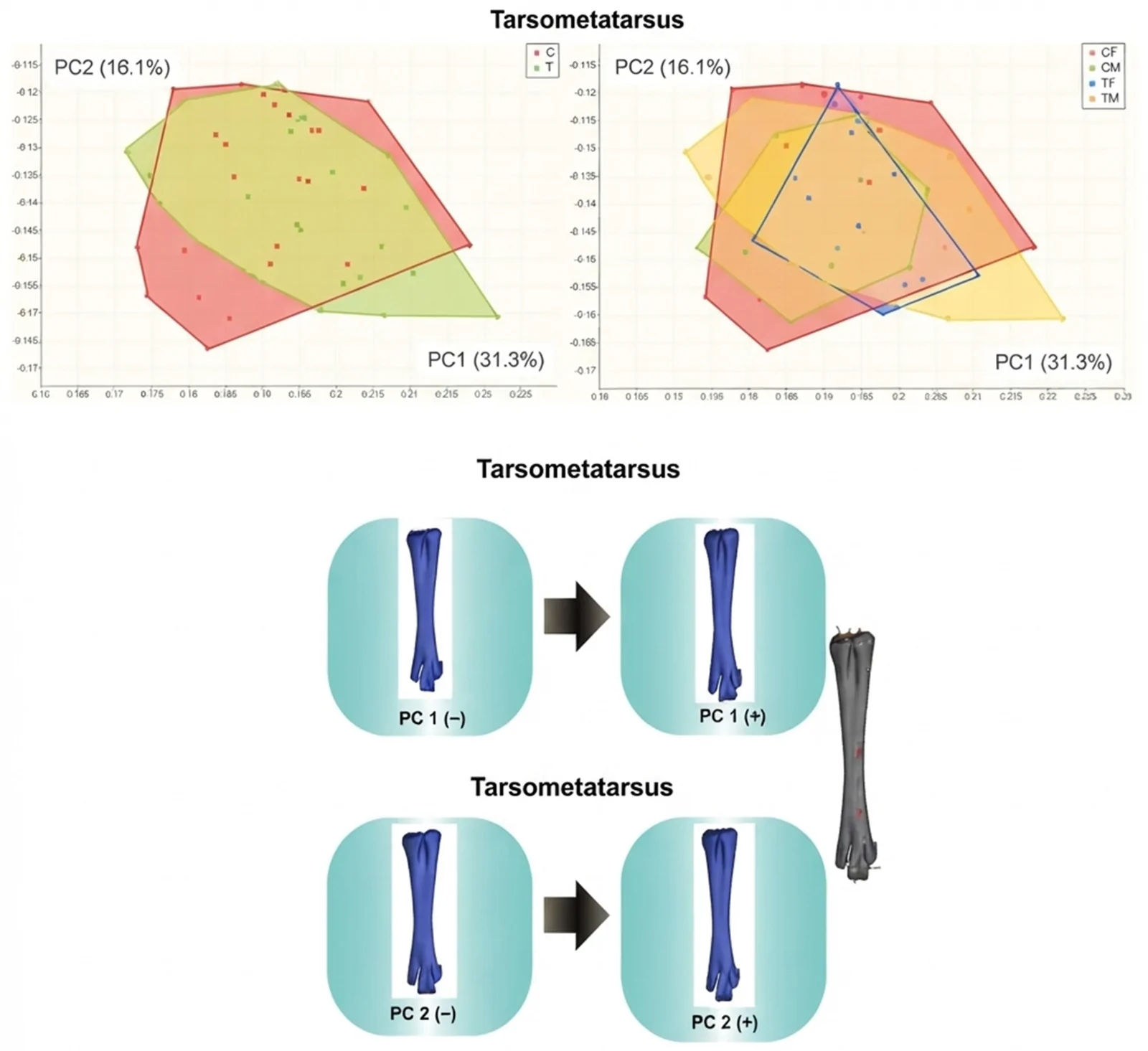
Principal component analysis (PCA) of tarsometatarsus shape variation obtained from three-dimensional geometric morphometric analysis. The left graph at the top: Two-group comparisons showing the control (C, red) and treatment (T, green) groups for the respective bone. Right graph at the top: Four-group subdivisions that separate the groups associated with the relevant bone by gender: control-female (CF, red), control-male (CM, green), treatment-female (TF, blue), and treatment-male (TM, yellow). The colored polygons represent the scatter plots of each group’s observations within the PCA plot. Shape changes associated with the positive direction of PC1 and PC2 are illustrated adjacent to each axis. Percentages indicate the proportion of total shape variation explained by each principal component.

**Table 1:** Analyzed composition of finisher diets on a fed basis.

Parameters	Control Diet	PSPA Diet
Dry matter (%)	90.23	90.42
Crude protein (%)	20.89	21.00
Ether extract (%)	7.95	7.87
Crude ash (%)	4.91	4.93
Calcium (%)	0.85	0.88
Total phosphorus (%)	0.70	0.73
Metabolizable energy (kcal/kg)	3323	3318

PSPA: phytogenic feed additive containing extracts of *Passiflora incarnata* and *Stevia rebaudiana.*

**Table 2:** Description of the landmarks related to the anatomical region list of the bones.

Landmark Number	Landmark Type	Anatomical Region	Landmark Number	Landmark Type	Anatomical Region
Tibiotarsus			Femur		
1, 7	I	*Crista cnemialis lateralis*	1	I	*Fovea ligamentum capitis*
2	II	*The proximal origin landmark of the cranial crista cnemialis*	2	II	*Facies articularis antitrochanterica* midpoint
3	I	*Incisura tibialis*	3–8	II	*Collum femoris* circumference
4	I	*Caput fibulae*	9–12	I	*Trochanter femoris*
5	I	*Area interarticularis*	13–17	I	*Crista trochanteris*
6	I	The deepest part of the *retropatellar fossa*	18, 19	I	*Impressiones iliotroch*.
8	I	*Facies articularis medialis*	20–23	II	Medial edge of femur
9	II	The point located between the proximal and distal ends of the *cranial cnemial crest* and the most cranial point of the *cranial cnemial crest*	24–27	II	Lateral edge of femur
10	II	The distal origin landmark of the *cranial crista cnemialis*	28–32	I	*Condylus medialis*
11–15	II	Tibiotarsus medial edge	33–37	I	*Sulcus intercondylaris*
16–20	II	Tibiotarsus lateral edge	38–42	I	*Condylus lateralis*
21	I	*Epicondylus lateralis*	Ulna		
22	I	*Epicondylus medialis*	1	I	*Olecranon*
23–26	III	*Condylus lateralis*	2–4	I	*Cotyla dorsalis*
27–30	III	*Condylus medialis*	5–9	I	*Margo cranialis*
31, 32	I	*Pons supratendineus*	10–14	I	*Margo caudalis*
			15–18	I	*Margo interosseus (ulna)*
Tarsometatarsus			19–22	I	*Facies articularis ulnacarpalis*
1–5	I	*Cotyla lateralis*	Radius		
6–10	I	*Eminentia intercotylaris*	1–4	I	*Cotyla* *humeralis*
11–15	I	*Cotyla medialis*	5–9	I	*Margo dorsalis*
16	II	The central point of the *lateral trochleae of metatarsus IV*	10–14	I	*Margo ventralis*
17	II	The central point of the *medial trochleae of metatarsus IV*	15–18	I	*Margo interosseus*
18	II	The central point of the *lateral trochleae of metatarsus III*	19–21	I	*Depressio radialis*
19	II	The central point of the *medial trochleae of metatarsus III*	Humerus		
20	II	The central point of the *lateral trochleae of metatarsus II*	1–6	I	*Caput humeri*
21	II	The central point of the *medial trochleae of metatarsus II*	7	I	*Tuberculum dorsale*
22	I	*Eminentia intertrochlearis lateralis*	8–12	I	*Margo dorsalis*
23	I	*Eminentia intertrochlearis medialis*	13–17	I	*Margo ventralis*
24–28	I	*Crista plantaris lateralis*	18–20	I	*Condylus dorsalis*
29–33	I	*Crista plantaris medialis*	21–23	I	*Condylus ventralis*
			24, 25	I	*Epicondylus ventralis*
Carpometacarpus					
1–5	I	*Trochlea carpalis*	16–20	I	*Os metacarpale minus lateralis*
6–10	I	*Facies articularis ulnacarpalis*	21–25	I	*Phalanges digitalis majus*
11–15	I	*Os metacarpale majus medialis*	26–30	I	*Phalanges digitalis minoris*

**Table 3:** Total polyphenol content and HPLC-quantified individual phenolic compounds of PSPA.

Compound	Content (mg/kg)
Total polyphenol content	5841
HPLC-quantified individual phenolic compounds	
Vanillic acid	0.87
Caffeic acid	9.92
Vanillin	2.94
*p*-Coumaric acid	0.71
Luteolin	0.58
Apigenin	1.29

Total polyphenol content was determined using the Folin–Ciocalteu spectrophotometric method. Individual phenolic compounds were identified and quantified by HPLC using compound-specific reference standards and external calibration. Only compounds that could be individually identified and quantified under the applied HPLC conditions are presented; therefore, the sum of the listed compounds does not represent the total polyphenol content.

**Table 4:** Volatile compounds identified in PSPA (% of total volatile composition).

Volatile Compound	Relative Abundance (%)	Volatile Compound	Relative Abundance (%)
Acetophenone	4.1	α-Humulene	0.5
Camphene	0.1	Geranylacetone	0.4
n-Propylbenzene	0.5	(E)-β-Farnesene	1.4
β-Pinene	0.5	Butylated hydroxyanisole (BHA)	1.0
1-Octen-3-ol	0.1	Alloaromadendrene	0.7
6-Methyl-5-hepten-2-one	0.2	Guaiyl acetate	0.5
2-Pentylfuran,	0.5	α-Muurolene	0.4
p-Cymene	1.7	β-Bisabolene	0.6
Limonene	0.3	γ-Cadinene	0.7
Phenylacetaldehyde	0.4	δ-Amorphene	1.0
m-Tolualdehyde	34.7	α--Calacorene	0.8
2-methylbenzaldehyde	9.9	Globulol	0.5
Linalool	0.6	Nerolidol, (E-)	1.0
Isosylvestrene	0.3	Spathulenol	7.6
Isoborneol	0.5	Ethyl laurate	1.4
Trans-2-nonenal	1.0	Thujopsan-2-α-ol	0.4
Terpinen-4-ol (4-carvomenthenol)	0.2	epi-α-Muurolol	1.8
α-Terpineol	1.0	Valeranone	0.9
Methylsalicylate	0.3	Caryophyllene oxide	1.1
Safranal	0.3	Longifolene	0.5
Thymoquinone	1.8	Tetrahydroionol	0.3
Cinnamaldehyde	0.3	Hexahydrofarnesyl acetone	1.1
Carvacrol	1.2	(E)-Sesquilavandulol	0.3
2-methoxy-4-vinylphenol	2.5	Sandracopimara-8(14), 15-diene	0.8
5-Pentyl-2-(5H)-furanone	0.5	Phytol	2.2
Eugenol	0.3	Isophytol	0.7
Valencene	1.4	Ambrettolide	6.1

**Table 5:** Effects of dietary PSPA supplementation and sex on carcass weight and carcass yield of broilers.

Source of Variation	Category	Carcass Weight (g)		Carcass Yield (%)	
		LS Mean ± SEM		LS Mean ± SEM	
Group	Control	2079.9 ± 8.16		73.9 ± 0.26	
	PSPA	2118.6 ± 8.09		74.4 ± 0.27	
Sex	Male	2158.0 ± 8.87		75.3 ± 0.26	
	Female	2040.4 ± 9.36		72.9 ± 0.27	
Group × sex	Control × Male	2141.6 ± 10.79		75.2 ± 0.37	
	Control × Female	2018.2 ± 14.02		72.5 ± 0.37	
	PSPA × Male	2174.5 ± 12.65		75.4 ± 0.36	
	PSPA × Female	2062.6 ± 11.17		73.3 ± 0.39	
Type III tests of fixed effects					
Fixed effect		Carcass weight		Carcass yield	
		F	*p* value	F	*p* value
Group		9.92	0.002	1.81	0.180
Sex		63.06	<0.001	41.17	<0.001
Group × Sex		0.29	0.590	0.42	0.516
Pre-slaughter live weight ^1^		1235.12	<0.001	-	-

Values are presented as least-squares means (LS means) ± standard error of the mean (SEM). The groups consisted of the Control and PSPA groups. Type III tests of fixed effects were obtained from the mixed linear model. ^1^ Pre-slaughter live weight was included as a covariate in the analysis of carcass weight only; carcass yield (%) was analyzed without adjustment for pre-slaughter live weight.

**Table 6:** Effects of dietary PSPA supplementation on carcass quality traits evaluated by house-level exact permutation and beta-binomial regression analyses.

Carcass Quality Trait	Control (%)	PSPA (%)	Difference (Percentage Points)	Exact Permutation Test (*p*-Value)
A-grade carcasses	62.62 ± 2.63 (60.10–65.35)	66.43 ± 1.94 (64.20–67.65)	+3.82	0.200
Broken wing	18.05 ± 2.03 (16.00–20.05)	17.55 ± 1.23 (16.25–18.70)	−0.50	0.800
Breast defect	5.06 ± 1.04 (4.35–6.25)	4.27 ± 0.75 (3.50–5.00)	−0.79	0.400
Thigh defect	7.48 ± 1.29 (6.00–8.35)	5.23 ± 1.33 (4.30–6.75)	−2.25	0.200
Bloody wing	5.80 ± 0.46 (5.30–6.20)	5.77 ± 0.63 (5.05–6.20)	−0.03	1.000
Other carcass defects	0.99 ± 0.09 (0.90–1.08)	0.75 ± 0.17 (0.65–0.95)	−0.24	0.200

Values are based on house-level percentages and are presented as the unweighted mean ± standard deviation (SD), with the range shown in parentheses (three independent houses per treatment group). The house was considered the experimental unit. Differences are expressed in percentage points and were calculated as the mean house-level percentage in the PSPA group–control group. Between-group comparisons were performed using two-sided exact permutation tests at the house level, with treatment labels permuted across the six independent houses. Exact *p*-values were calculated from the complete permutation distribution.

**Table 7:** Effects of dietary PSPA supplementation and sex on the proximate composition of breast meat and liver (% as-is basis).

Variable	Group		Significance (*p*-Value)		
	Control	PSPA	Group	Sex	Group × Sex
Breast meat					
Dry matter	26.45 ± 0.07	26.61 ± 0.07	0.123	0.167	0.389
Crude protein	24.09 ± 0.08	24.24 ± 0.08	0.154	0.281	0.429
Crude fat	1.18 ± 0.06	1.18 ± 0.06	0.971	0.824	0.958
Ash	1.18 ± 0.06	1.18 ± 0.06	0.979	0.844	0.949
Liver					
Dry matter	28.06 ± 0.22	28.39 ± 0.22	0.282	0.375	0.467
Crude protein	21.14 ± 0.13	21.29 ± 0.13	0.401	0.906	0.972
Crude fat	5.41 ± 0.16	5.59 ± 0.16	0.434	0.144	0.386
Ash	1.51 ± 0.04	1.51 ± 0.04	0.897	0.094	0.592

Values are presented as least-squares means (LS means) ± standard error of the mean (SEM). The groups consisted of the Control and PSPA groups. Least-squares means and Type III tests of fixed effects were obtained from linear mixed-effects models fitted using restricted maximum likelihood (REML). Group, sex, and their interaction were included as fixed effects, and house was included as a random intercept to account for clustering of birds within houses.

**Table 8:** Effects of dietary PSPA supplementation and sex on serum inflammatory cytokines, corticosterone, and oxidative stress biomarkers.

Variable	Group		SEM	Significance (*p*-Value)		
	Control	PSPA		Group	Sex	Group × Sex
IL-1β ^1^ (pg/mL)	32.29 (26.68–39.08)	22.55 (18.62–27.29)	—	0.021	0.084	0.972
IL-6 ^1^ (pg/mL)	83.38 (77.84–89.13)	56.88 (53.09–60.82)	—	<0.001	0.307	0.114
TNF-α ^1^ (pg/mL)	31.05 (25.12–38.47)	20.37 (16.49–25.18)	—	0.018	0.274	0.822
IL-10 (pg/mL)	187.24 (158.2–216.3)	228.71 (199.7–257.7)	11.46	0.048	0.935	0.846
Corticosterone (ng/mL)	43.18 (37.41–48.95)	34.16 (28.39–39.93)	2.083	0.037	0.755	0.869
TAS (mmol Trolox Equivalent/L)	1.33 (1.25–1.41)	1.34 (1.26–1.42)	0.04	0.858	0.942	0.723
TOS (µmol H_2_O_2_ Equivalent/L)	14.41 (12.10–16.72)	11.09 (8.78–13.41)	0.84	0.048	0.678	0.911
OSI	1.096 (0.955–1.237)	0.837 (0.695–0.978)	0.051	0.022	0.754	0.957

*n* = 30. Values are presented as least-squares means (LS means). Values in parentheses indicate the 95% confidence interval (95% CI). The SEM column represents the standard error of the LS mean. ^1^ IL-1β, IL-6, and TNF-α were analyzed after log10 transformation and are presented as back-transformed geometric LS means (95% CI). All remaining variables are presented on the original measurement scale. Data were analyzed using linear mixed-effects models fitted by restricted maximum likelihood (REML), with treatment, sex, and treatment × sex included as fixed effects and house included as a random intercept.

**Table 9:** Multivariate analysis of variance (MANOVA) for bone shape differences between the groups and sexes based on three-dimensional geometric morphometrics.

Bone	Group (Control vs. PSPA)			Sex (Female vs. Male)		
	Wilks’ λ	F	*p*-Value	Wilks’ λ	F	*p*-Value
Humerus	0.8514	0.7155	0.7050	0.8197	1.383	0.2366
Radius	0.8415	0.9684	0.4687	0.8788	0.7093	0.6643
Ulna	0.9158	0.5910	0.7597	0.7027	2.720	0.0193
Carpometacarpus	0.7692	1.972	0.0797	0.7340	2.382	0.0364
Femur	0.7499	2.287	0.0429	0.9221	0.5792	0.7693
Tibiotarsus	0.8683	1.105	0.3741	0.6204	4.458	0.0006
Tarsometatarsus	0.8466	1.217	0.3126	0.8587	1.105	0.3756

Values are derived from a MANOVA performed based on all PC scores. Wilks’ lambda (λ), F-statistic, and associated *p*-values are presented for comparisons between treatment groups and between sexes. *p*-values are FDR-adjusted using the Benjamini–Hochberg procedure; FDR-adjusted *p* < 0.05 was considered statistically significant.

**Table 10:** MANOVA comparing bone shape among the groups of Control Female, Control Male, PSPA Female, and PSPA Male.

Bone	Wilks’ λ	F	*p*-Value
Humerus	0.5223	0.9508	0.5460
Radius	0.5639	1.032	0.4336
Ulna	0.5454	1.388	0.1365
Carpometacarpus	0.4546	1.909	0.0154
Femur	0.5856	1.294	0.1903
Tibiotarsus	0.4512	2.148	0.0046
Tarsometatarsus	0.5740	1.318	0.1754

Values are derived from MANOVA comparing the four experimental groups (Control Female, Control Male, PSPA Female, and PSPA Male) based on all PC scores. Wilks’ lambda (λ), F-statistic, and associated *p*-values are presented for comparisons between treatment groups and between sexes. *p*-values are FDR-adjusted using the Benjamini–Hochberg procedure; FDR-adjusted *p* < 0.05 was considered statistically significant.

**Table 11:** Pairwise MANOVA comparisons among the groups based on bone shape (*p*-values).

Bone	CF–CM	CF–TF	CF–TM	CM–TF	CM–TM	TF–TM
Humerus	0.7974	0.9664	0.6853	0.7075	0.4331	0.4413
Radius	0.5773	0.2690	0.6831	0.7628	0.7171	0.4956
Ulna	0.7398	0.9756	0.1247	0.4044	0.3167	0.0396
Carpometacarpus	0.0645	0.5083	0.5877	0.0199	0.1095	0.4544
Femur	0.8514	0.0788	0.4764	0.2580	0.5612	0.4183
Tibiotarsus	0.3772	0.3454	0.0842	0.0143	0.5136	0.0061
Tarsometatarsus	0.2904	0.3327	0.4935	0.7532	0.2893	0.2387

CF, Control Female; CM, Control Male; TF, PSPA Female; TM, PSPA Male. Values represent *p*-values obtained from pairwise multivariate comparisons based on the PCA-derived PC scores. *p*-values are FDR-adjusted using the Benjamini–Hochberg procedure; FDR-adjusted *p* < 0.05 was considered statistically significant.

## Data Availability

The data that support the findings of this study are available from the corresponding author upon reasonable request.

## Abbreviations

The following abbreviations are used in this manuscript:

PSPA	A phytogenic additive containing *Passiflora incarnata* and *Stevia rebaudiana* extracts
HPA	Hypothalamic–pituitary–adrenal
ME	Metabolizable energy
IRIS	Intelligent Reporting, Inspection and Selection
TAS	Total antioxidant status
TOS	Total oxidant status
OSI	Oxidative stress index
CT	Computed tomography
GPA	Generalized Procrustes Analysis
PCA	Principal component analysis
MANOVA	Multivariate analysis of variance
PC	Principal component
